# Definitive external-beam radiotherapy versus radical prostatectomy in clinically localized high-risk prostate cancer: a retrospective study

**DOI:** 10.1186/s12894-018-0432-6

**Published:** 2019-01-05

**Authors:** Fundagul Andic, Volkan Izol, Serkan Gokcay, Hasan Suat Arslantas, Yildirim Bayazit, Hatice Coskun, Mustafa Zuhtu Tansug, Yasar Sertdemir

**Affiliations:** 1 Faculty of Medicine, Department of Radiation Oncology, Cukurova University, Adana, Turkey; 2 Faculty of Medicine, Department of Urology, Cukurova University, Adana, Turkey; 3 Department of Medical Oncology, Mehmet Akif Inan Training and Research Hospital, Sanliurfa, Turkey; 4 Faculty of Medicine, Department of Radiation Oncology, Gaziantep University, Gaziantep, Turkey; 5 Faculty of Medicine, Department of Biostatistics, Cukurova University, Adana, Turkey; 6 Balcali Hastanesi, Radyasyon Onkolojisi AD, Cukurova Universitesi, 01330 Sarıcam, Adana, Turkey

**Keywords:** High-risk prostate cancer, Radiotherapy, Radical prostatectomy, Treatment outcomes

## Abstract

**Background:**

Optimal treatment of high-risk prostate cancer remains controversial. We aimed to compare treatment outcomes of prostate cancer patients treated with definitive external-beam radiotherapy (ExRT) or radical prostatectomy (RP).

**Methods:**

The records of 120 high-risk clinical stage T2b-T4 N0 M0 prostate cancer patients treated with definitive ExRT or RP were reviewed. Patients with pretreatment prostate-specific antigen (PSA) levels ≥20 ng/mL or clinical ≥T3 stage or Gleason score (GS) ≥8 were included in the study. Biochemical failure free survival (BFFS), distant metastasis free survival (DMFS), cancer-specific survival (CSS) and overall survival (OS) were analyzed. Cox regression analysis was performed to determine predictors of BF.

**Results:**

Seventy-two patients received definitive ExRT with androgen-deprivation therapy in 95.8% and 48 patients underwent RP with pelvic lymph node dissection. Mean age (67.7 ± 6.6 vs 64.5 ± 7.6 year, *p* = 0.017) and the rate of patients with PSA levels ≥20 ng/mL (69.4% vs 47.9%, *p* = 0.022) were higher in the definitive ExRT group than the RP group. Distributions of GS and clinical T stage were similar. Mean follow-up was 60.2 ± 30.3 months in the definitive ExRT group and 41.3 ± 21.5 months in the RP group (*p* <  0.001). Twenty-five % of the RP group received adjuvant ExRT and 41.7% received salvage ExRT. Biochemical failure was significantly higher (52.1% vs 21.4%, *p* <  0.001) and the mean BFFS was significantly lesser (34.4 ± 3.9 vs 97.8 ± 5.9 months, *p* < 0.001) in the RP group than the definitive ExRT group. However, DMFS, CSS and OS were similar in both groups. In multivariate analysis, being in the RP group significantly increased the risk of BF (*p* < 0.001). Furthermore, not receiving pelvic lymphatic irradiation in the definitive ExRT group (*p* = 0.048) and having positive surgical margin in the RP group (*p* = 0.050) increased the risk of BF.

**Conclusions:**

BF was significantly higher and the mean BFFS was significantly lesser in high-risk prostate cancer patients undergoing RP than definitive ExRT while DMFS, CSS and OS were similar in both treatment groups.

## Background

High-risk disease accounts approximately 30% of prostate cancer (PCa) [1, 2]. However, the current definitions of high-risk PCa include a heterogeneous group of patients with a range of prognoses and the optimal management of the subgroups is under debate [3–7].

Local treatment of high-risk PCa provides local control and also prevents further seeding of distant metastatic sites. Conventionally, high-risk PCa patients are treated with a combination of definitive external-beam radiotherapy (ExRT) and androgen-deprivation therapy (ADT) [8–10]. However, recent published data suggests that radical prostatectomy (RP) shows excellent local tumor control and similar oncological results in high-risk PCa patients, especially in combination with multimodal treatments involving ADT and radiotherapy [11–14]. Consequently, the American Urological Association (AUA) and European Association of Urology (EAU) support RP with extended pelvic lymph node dissection (PLND) as an optional treatment for a selective group of patients in the context of multimodal treatment [3, 4].

While outcomes for definitive ExRT and RP have been published independently these treatments have not been compared prospectively and the optimal management of high-risk PCa is still controversial. However, side effect profiles of definitive ExRT and RP might play a role in the decision of treatment. Treatment specific quality of life studies in clinically localized PCa demonstrate that sexual function and urinary control are better after ExRT than RP and gastro-intestinal side effects are more common after ExRT [15, 16]. Furthermore, adverse events due to combination of radiotherapy with long-term ADT, such as heart failure, arrhythmia, liver dysfunction, glucose intolerance, hot flush and diabetes may pose additional problems [17, 18].

In this single-institution retrospective study, we aimed to analyze the treatment outcomes of high-risk PCa patients either treated with definitive ExRT or RP as first-line treatment of the disease.

## Methods

This study is approved by the Institutional Ethics Review Board.

The records of 120 patients with clinical stage T2b-T4 N0 M0 high-risk PCa who were treated with definitive ExRT or RP with PLND and/or followed at the Departments of Radiation Oncology and Urology between August 2007 and March 2018 were analyzed retrospectively. Patients were staged according to the seventh edition of the American Joint Committee on Cancer Classification and Staging of Tumors [19].

Eligibility criteria included: untreated, high-risk, clinical node negative, none-metastatic, histologically confirmed adenocarcinoma of the prostate. Patients at least having one of the following factors at pretreatment diagnostic evaluation: prostate-specific antigen (PSA) level ≥ 20 ng/mL, clinical ≥T3 stage and Gleason score (GS) ≥8 were accepted as high-risk PCa as defined in the AUA guideline [3].

All patients were evaluated with complete history, physical examination and blood PSA counts. Pelvic computed tomography (CT) and or magnetic resonance imaging, and bone scan were used for staging. Additionally, positron emission tomography (PET)/CT scan or prostate-specific membrane antigen PET /CT were applied in some patients as a part of their workup.

Follow-up of asymptomatic patients included disease specific history and blood PSA measurement at least 3 times (at three, six and twelve months) in the first year after treatment, then every six months until three years, and then annually. Restaging work-up was done if necessary. Examination intervals were adjusted individually at biochemical and clinical progression. Patient follow-up was completed through the review of medical records and phone calls from the patients.

In patients who underwent RP, a detectable or rising PSA value after surgery that is ≥0.2 ng/ml with a second confirmatory level is accepted as biochemical failure (BF) and the date of BF is accepted as the first rise (≥0.2 ng/mL) in PSA [20]. In patients receiving definitive ExRT, a PSA rise of ≥2 ng/mL above the nadir PSA after radiotherapy is accepted as BF and the date of BF is defined by the time the rise in PSA (≥2 ng/mL above the nadir) [21].

Postoperative ExRT was accepted as adjuvant if ExRT was started before BF without local and distant metastatic disease within 6 months after RP and accepted as salvage if ExRT was started after local and/or BF without distant metastatic disease. The decision of adjuvant or salvage ExRT and/or ADT were made by an urologist in cooperation with a radiation oncologist. The patients with positive surgical margins and positive lymph nodes were mostly treated with adjuvant ExRT. However, the patients with negative surgical margins and negative lymph nodes were most regularly followed-up and were further treated if they concluded with clinical failure.

Following definitive treatment, patients who have only BF with or without local recurrence are assessed for salvage local therapy often combined with ADT and patients who have subsequent metastatic disease received docetaxel or abiraterone acetate in combination with ADT.

Upgrading and downgrading of GS were defined as an increase or decrease, respectively, from one prognostic risk group to another, similar to upstaging and downstaging of T stage. Based on the AUA guideline, prognostic risk groups for GS were defined as follows: GS ≤6 (low risk), GS 7 (intermediate risk), and GS ≥8 (high risk) and prognostic risk groups for T stage were defined as follows: ≤T2a (low risk), T2b-T2c (intermediate risk) and ≥ T3 stage (high risk) [3].

### End points

Mean follow-up was 60.2 ± 30.3 months in the definitive ExRT group and 41.3 ± 21.5 in the RP group. The primary end point was the rate of BF and BF free survival (BFFS). Additionally, distant metastases free survival (DMFS), cancer-specific survival (CSS) and overall survival (OS) were analyzed.

Possible predictive risk factors for BF were defined as age, pretreatment PSA level ≥ 20 ng/mL, GS ≥8 at biopsy and RP specimen, clinical and pathological ≥T3 tumor, pathological N1 stage, positive surgical margins, pelvic lymphatic irradiation (PLI) and short term (< 2 year) ADT use.

In the definitive ExRT group, in order to analyze whether duration of ADT may predict BF or not, ADT duration was calculated from the day of initiation of ADT to the last day of ADT effectiveness in cases without BF and to the day of BF in cases with BF.

### Statistical analysis

All analyses were performed using IBM SPSS Statistics for Windows Version 19.0 statistical software package (Armonk, NY: IBM Corp). Categorical variables were expressed as numbers and percentages, whereas continuous variables were summarized as mean and standard deviation and as median and minimum-maximum where appropriate. Chi-square/Fisher Exact test was used to compare categorical variables between groups. For comparison of continuous variables between two groups, the Student’s t-test or Mann-Whitney U test was used depending on whether the statistical hypotheses were fulfilled or not. Kaplan-Meier method and log rank test was performed for survival analysis. Cox regression analysis was performed to determine significant predictors of BF. Results of univariate and multivariate Cox regression analysis are given with odds ratio and 95% confidence interval. The statistical level of significance for all tests was considered to be 0.05.

## Results

Seventy-two (60%) patients underwent definitive ExRT and 48 (40%) patients underwent RP with PLND as first-line therapy of high-risk PCa. The characteristics of high-risk PCa patients are shown in Table 1. No difference with regard to pretreatment clinical T stage and GS was found between the treatment groups. However, pretreatment mean PSA levels (*p* = 0.049) and the rate of patients with pretreatment PSA levels ≥20 ng/mL (*p* = 0.022) were significantly higher in the definitive ExRT group than the RP group.

**Table 1:** Characteristics of patients

	ExRT (*n* = 72)	RP (*n* = 48)	*p*
Age (years)	67.7 ± 6.6 67.2 (43.9–80.1)	64.5 ± 7.6 65.3 (47.3–79.9)	0.017
Follow-up (months)	60.2 ± 30.3 53.7 (10.3–125.8)	41.3 ± 21.5 37.8 (9.6–128.9)	< 0.001
Pretreatment PSA (ng/mL)	34.6 ± 30.7 25.4 (3.4–172)	27.2 ± 26.3 17 (3.2–138.7)	0.049
Pretreatment PSA ≥20 ng/mL n (%)	50 (69.4)	23 (47.9)	0.022
Clinical T stage n (%)			
T2	46 (63.9)	35 (72.9)	0.474
T3	22 (30.6)	12 (25.0)	
T4	4 (5.6)	1 (2.1)	
Gleason score (biopsy) n (%)			
6	17 (23.6)	8 (16.7)	0.636
7	15 (20.8)	10 (20.8)	
> 8	40 (55.6)	30 (62.5)	

*ExRT*, external-beam radiotherapy; *RP*, radical prostatectomy; *PSA*, prostate-specific antigen

Sixty-nine (95.8%) of the patients receiving definitive ExRT also received ADT in combination with ExRT as first-line treatment. ADT was not prescribed in three patients because of their cardiovascular comorbidities. While 65.3% (*n* = 47) of the patients receiving definitive ExRT received ADT ≥2-years, 25% (*n* = 18) of them received ≥1-year to < 2-year and 5.6% (*n* = 4) of them received less than < 1-year.

All patients had RP with extended PLND. However, 3 patients underwent surgery at an outside facility. Pathological characteristics of RP are given in Table 2. Forty-four out of 48 (91.7%) patients who underwent RP had adverse pathological risk factors (≥T3 tumor and/or positive surgical margins and/or positive lymph nodes and or GS ≥8).

**Table 2:** Pathological characteristics of radical prostatectomy

Extended PLND n (%)	48 (100)
Removed LN	13.3 ± 7.4 12 (5–38)
N1 stage n (%)	16 (33.3)
Positive surgical margin n (%)	28 (58.3)
Gleason score n (%)	
6	2 (4.2)
7	19 (39.6)
≥ 8	27 (56.3)
Gleason score upgrading n (%)	8 (16.7)
Gleason score downgrading n (%)	7 (14.6)
T stage n (%)	
T2	9 (18.8)
T3	38 (79.2)
T4	1 (2.1)
T stage upgrading n (%)	25 (52.1)
T stage downgrading n (%)	0 (0)

*PLND:* pelvic lymph node dissection; *LN*: lymph nodes

Characteristics of definitive, adjuvant and salvage ExRT are given in Table 3. Three-dimensional conformal radiation therapy (3D-CRT) or intensity modulated radiation therapy (IMRT) techniques were used in all 104 patients receiving ExRT. However, 11.5% of the patients (5 in definitive ExRT group, 4 in adjuvant ExRT group and 3 in salvage ExRT group) received their ExRT at an outside facility.

**Table 3:** Characteristics of external-beam radiotherapy

	Definitive ExRT (*n* = 72)	Adjuvant ExRT (*n* = 12)	Salvage ExRT (*n* = 20)
Volume	P + SV +/− PLI	Operation bed +/− PLI	Operation bed +/− PLI
Dose*	74 Gy (70–76)	66 Gy (60–70.2)	66 Gy (60–74)
PLI n (%)	18 (25.0)	3 (33.3)	8 (40)
Technique n (%)			
3D-CRT	65 (90.3)	10 (83.3)	18 (90)
IMRT	7 (9.7)	2 (16.7)	2 (10)

*ExRT*, external-beam radiotherapy; *P*, prostate; *SV*, seminal vesicle; *PLI*, pelvic lymphatic irradiation; *3D-CRT*, three-dimensional conformal radiotherapy; *IMRT*, intensity modulated radiotherapy

*, radiotherapy was given with 1.8–2 Gy daily fraction dose

In the RP group, 12 (25%) patients received adjuvant ExRT and 20 (41.7%) patients received salvage ExRT for local and/or BF. Furthermore, 9 of the patient in adjuvant ExRT group and 12 of the patients in salvage ExRT group also received ADT in combination with ExRT.

Prior to salvage ExRT, median PSA level of totally 20 patients was 1.09 (0.25–43.5). Salvage ExRT was administered at a PSA level of < 0.5 ng/mL in 3 (15%) patients, at a PSA level of 0.5 to1.5 in 10 (50%) patients and > 1.5 ng/mL in 7 (35%) patients.

Risk groups of GS were confirmed on RP specimen in 33 (68.8%) out of 48 patients who underwent RP. Eight patients (16.7%) had upgrading on RP specimen: 4 patients upgraded from a low risk GS 6 to an intermediate risk GS 7, 2 patients from a low risk GS 6 to a high risk GS 9 and 2 patients from an intermediate risk GS 7 to a high risk GS 8. Furthermore, 7 (14.6%) patients had downgrading on RP specimen from a high risk GS 8 to an intermediate risk GS 7.

Risk groups of clinical T stages were confirmed on RP specimen in 23 (47.9%) out of 48 patients. However, 25 (52.1%) patients had upstaging on RP specimen from a T2b-T2c stage to a T3 stage in 24 patients and from a T2c to a T4 stage in 1 patient.

**Fig. 1:**
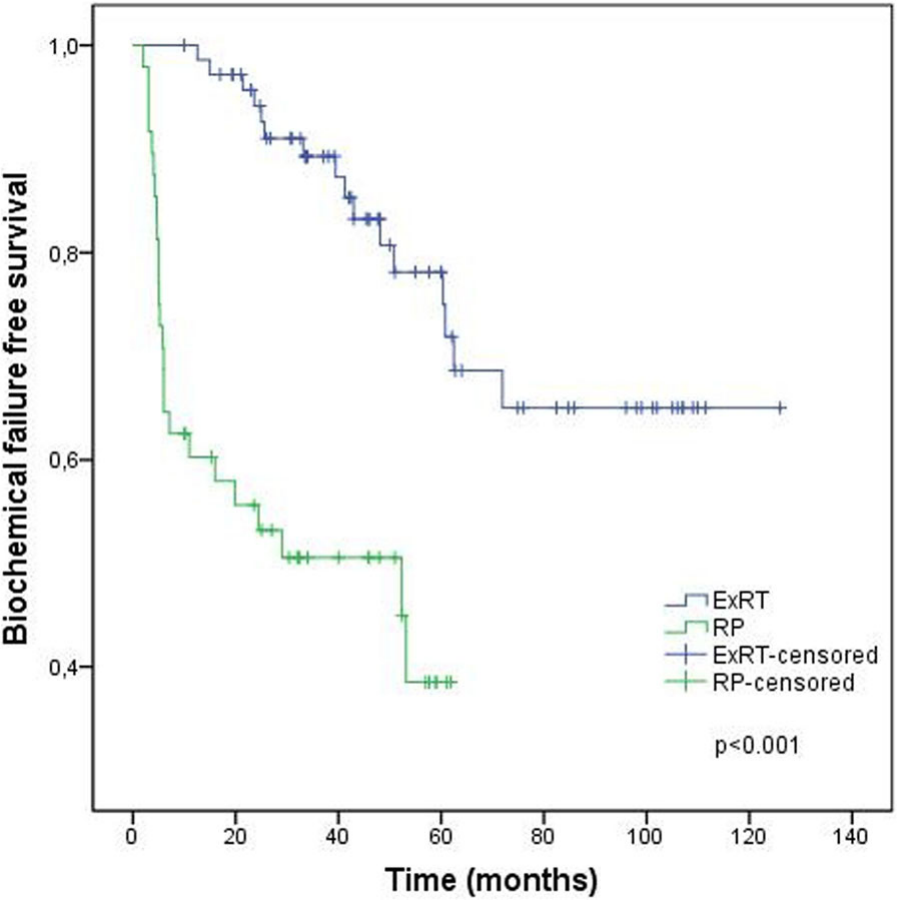
Biochemical failure free survival. ExRT, external-beam radiotherapy; RP, radical prostatectomy

**Fig. 2:**
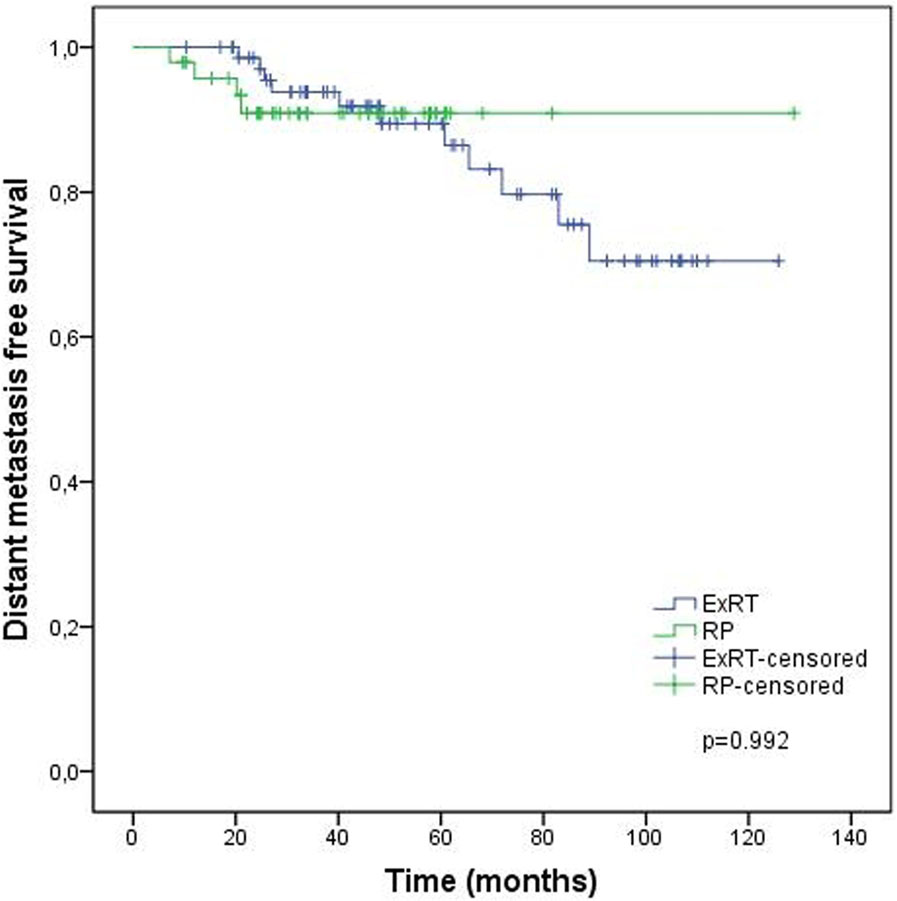
Distant metastasis free survival. ExRT, external-beam radiotherapy; RP, radical prostatectomy

**Fig. 3:**
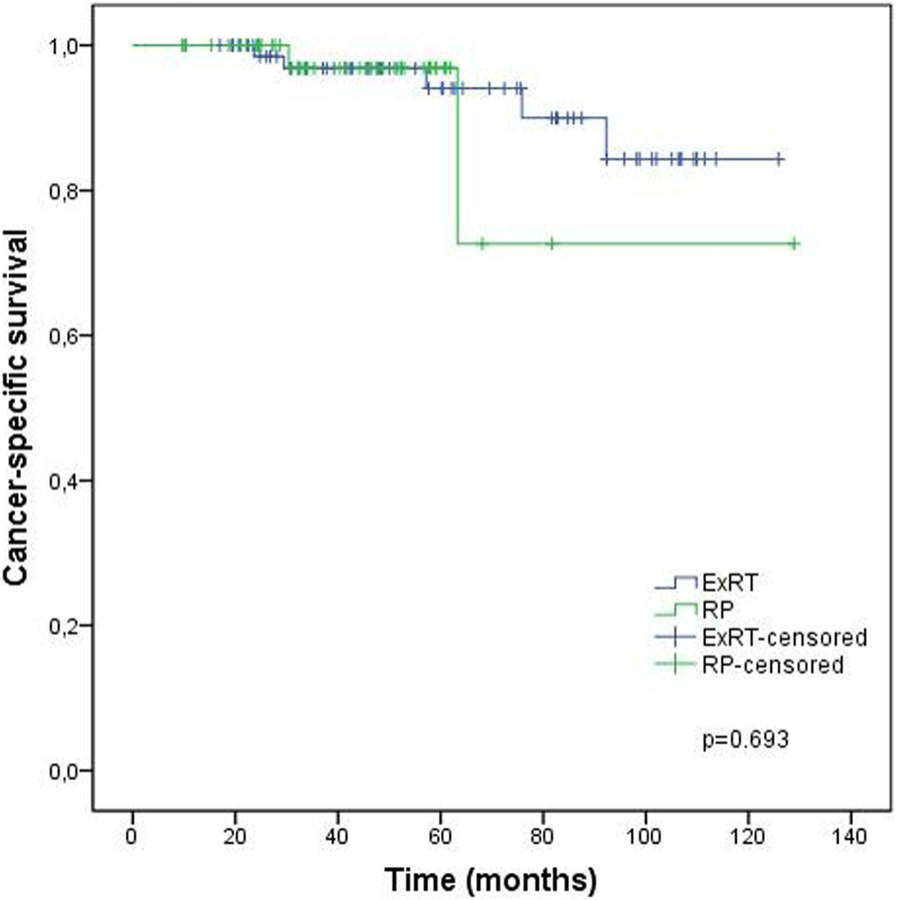
Cancer-specific survival. ExRT, external-beam radiotherapy; RP, radical prostatectomy

**Fig. 4:**
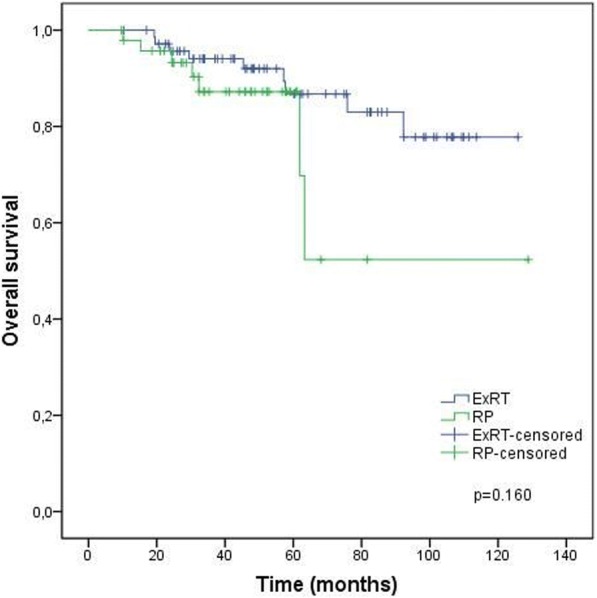
Overall survival. ExRT, external-beam radiotherapy; RP, radical prostatectomy

Treatment outcomes according to the treatment groups are given in Figs. 1, 2, 3 and 4 and Table 4. BF was significantly higher (52.1% vs 21.4%, *p* < 0.001) and the mean BFFS was significantly lesser (34.4 ± 3.9 vs 97.8 ± 5.9 months, p < 0.001) in the RP group than the definitive ExRT group. However, distant metastasis, cancer-specific mortality, all-cause mortality, DMFS, CSS and OS were similar in both groups.

**Table 4:** Outcomes of treatment groups

Treatment Outcomes	Definitive ExRT (*n* = 72)	RP (*n* = 48)	p
BF n (%)	16 (21.4)	25 (52.1)	< 0.001
Mean BFFS (months)	97.8 ± 5.9	34.4 ± 3.9	< 0.001
3 year BFFS (%) (95% CI)	89.3 (95 81.7–96.9)	50.6 (36.1–65.1)	
5 year BFFS (%) (95% CI)	78.1 (66.7–89.5)	38.5 (20.1–56.9)	
DM n (%)	11 (15.3)	4 (8.3)	0.399
Mean DMFS (months)	106.6 ± 5.1	118.5 ± 4.9	0.992
3-year DMFS (%) (95% CI)	93.8 (87.9–99.7)	90.9 (82.4–99.4)	
5-year DMFS (%) (95% CI)	89.5 (81.4–97.6)	90.9 (82.4–99.4)	
Cancer-specific mortality n (%)	5 (6.9)	2 (4.2)	0.701
Mean CSS (months)	116.9 ± 5.1	109.9 ± 14.0	0.693
3 year CSS (%)	96.8 (92.5–100.0)	96.9 (90.8–100.0)	
5 year CSS (%)	94.1 (87.2–100.0)	96.9 (90.8–100.0)	
All-cause mortality n (%)	9 (12.5)	7 (14.6)	0.788
Mean OS (months)	110.9 ± 4.6	92.3 ± 13.4	0.160
3 year OS (%) (95% CI)	94.1 (88.4–99.7)	87.2 (76.6–97.9)	
5 year OS (%) (95% CI)	86.8 (77.2–96.3)	87.2 (76.6–97.9)	

*ExRT*, external-beam radiotherapy; *RP*, radical prostatectomy; *BF*, biochemical failure; *BFFS*, biochemical failure free survival; *DM*, distant metastasis; *DMFS*, distant metastasis free survival; *CSS*, cancer-specific survival; *OS*, overall survival

Results of the univariate Cox regression analyses for potential predictors of BF are given in Table 5. When all patients were included, univariate analysis showed that being in the treatment group RP was the only significant (p < 0.001) predictor of BF while age was borderline significant (*p* = 0.066) and multivariate analysis showed that being in the treatment group RP was still the only significant (*p* < 0.001) predictor of BF and pretreatment PSA level ≥ 20 ng/mL was borderline significant (*p* = 0.069). When only definitive ExRT treatment group was included, univariate analysis showed that not receiving PLI was borderline significant (*p* = 0.071) to predict BF and multivariate analysis showed that after risk adjustment according to ≥2-year ADT use, BF risk was significantly higher in patients who did not receive PLI (*p* = 0.048) than patients who received. When only RP treatment group was included, both univariate and multivariate analysis showed that positive surgical margin was the only significant (*p* = 0.050) predictor of BF.

**Table 5:** Possible predictive factors in univariate and multivariate analysis

	OR	95% CI	p
All patients-univariate			
Age	0.96	0.92–1.00	0.066+
PSA ≥20 ng/mL	1.26	0.66–2.41	0.480
Clinical ≥T3 stage	0.70	0.35–1.41	0.319
GS ≥8 at biopsy	1.29	0.69–2.42	0.430
Treatment group RP	4.61	2.36–9.00	< 0.001*
All patients-multivariate			
PSA ≥20 ng/mL	1.85	0.95–3.59	0.069+
Treatment group RP	5.35	2.69–10.61	< 0.001*
ExRT group-univariate			
ADT use < 2-year	1.98	0.72–5.49	0.188
No PLI	2.53	0.93–6.91	0.071+
ExRT group-multivariate			
ADT use < 2-year	2.42	0.85–6.89	0.098
No PLI	2.87	1.01–8.16	0.048*
RP group-univariate			
Positive surgical margin	2.41	1.00–5.78	0.050*
Pathological N1 stage	1.97	0.87–4.46	0.105
Pathological ≥T3 stage	2.17	0.65–7.32	0.210
GS ≥8 at RP specimen	1.83	0.80–4.18	0.154
RP group-multivariate			
Positive surgical margin#	2.41	1.00–5.78	0.050*

*OR*, Odds ratio; *CI*, confidence Interval; *PSA*, prostate specific antigen; *GS*, Gleason score; *RP*, radical prostatectomy; *ExRT*, external-beam radiotherapy; *ADT*, androgen deprivation treatment; *PLI*, pelvic lymphatic irradiation

*indicates statistically significant data; + indicates borderline significant data

#, for the RP group, positive surgical margin was the only significant risk factor predicting BF in multivariate analysis

## Discussion

There is no consensus on the best treatment for high-risk PCa patients. Options include definitive ExRT with ADT or RP with PLND followed by adjuvant radiotherapy in cases with unfavorable pathological features. The results of this study showed that the rate of BF was significantly higher (52.1% vs 21.4%, *p* < 0.001) and the mean BFFS was significantly lesser (34.4 ± 3.9 vs 97.8 ± 5.9 months, *p* < 0.001) in the RP group than the definitive ExRT group. Five-year BFFS was 38.5 and 78.1% in the RP and the definitive ExRT group, respectively. Furthermore, 5-year DMFS, CSS and OS were similar in both groups. In multivariate analysis, when all patients were included, being in the treatment group RP significantly increased the risk of BF (p < 0.001) and pretreatment PSA levels ≥20 ng/mL was borderline significant (*p* = 0.069); when only definitive ExRT group was included, after risk adjustment according to ≥2-year ADT use, not receiving PLI significantly increased the risk of BF (*p* = 0.048); and when only RP group was included, positive surgical margin was the only significant (*p* = 0.050) predictor of BF.

Suggestion of RP as a first-line treatment option in high-risk PCa patients is based on several large published retrospective series demonstrating favorable 10-year CSS rates ranging around 90 to 80% and 5-year BFFS rates ranging around 70 to 50% [12, 22–24]. However, such high survival rates and high BFFS rates may be associated with the definitions of high-risk disease and thus capturing the patients with more favorable high-risk disease. In our study, 5-year CSS rate was 96.9% and 5-year BFFS was 38.5% in the RP group.

Furthermore, several studies have retrospectively compared RP with ExRT in high-risk PCa patients. In a combined analysis including 1238 patients undergoing RP and 609 patients undergoing ExRT with or without ADT, 10-year CCS rates were 92, 92, and 88% for patients receiving RP, ExRT with ADT, and ExRT alone, respectively (*p* = 0.06) [25]. After multivariable risk adjustment, there was no significant difference in DMFS or CSS. However, the risk of all-cause mortality was greater after ExRT with ADT when compared to RP which could be explained by selection bias and contribution of potential adverse cardiovascular effects of ADT [25]. In our study 5-year DMFS and 5-year CCS was similar (89.5% vs 90.9 and 94.1% vs 96.9%) in the definitive ExRT and the RP groups, respectively, as well as the rate of all-cause mortality (86.8% vs 87.2%).

In a retrospective study from Italy reported outcomes of 288 patients with high-risk PCa either underwent ExRT (*n* = 162) in combination with nine months of ADT or underwent (*n* = 122) RP with pelvic lymph node sampling. Three-year BFFS favored ExRT targeted to prostate and seminal vesicle without PLI (86.8% vs 69.8%, *p* = 0.001) [26]. However, ADT duration was none-optimal and follow-up period was relatively short. In our study, 95.8% (*n* = 69) of the patients receiving definitive ExRT also received ADT in combination with ExRT as initial treatment. Furthermore, ADT duration was ≥2-years in 65.3% (*n* = 47) of the patients and ≥ 1-year to < 2-year in 25% (*n* = 18) of them.

Another retrospective study reported 7.8% absolute benefit in 8-year metastatic progression with RP compared to ExRT in a high-risk cohort of PCa patients [27]. However, omission of PLI and short term (3 to 6 months) ADT use in combination with ExRT and higher pretreatment risk factors (PSA, clinical stage, and GS) in the ExRT group than the RP group were the major limitations of the study [27]. In our study, 5-year DMFS were similar in both treatment groups. Furthermore, use of ≥2-years ADT in 65.3% and performing PLI in 25% of the definitive ExRT group, and similar distribution of pretreatment clinical stage and GS in both groups likely offered a healthier assessment in our study.

Based on three prospective randomized trials showing clinical or survival benefit for adjuvant radiotherapy in patients with pathological T3 disease or positive margins, adjuvant radiotherapy is recommended to PCa patients with high-risk of local recurrence when the PSA is undetectable (< 0.2 ng/mL) [28–31]. Typically, adjuvant radiotherapy is administered 3–6 months postoperatively when incontinence has stabilized or resolved. However, administration of adjuvant radiotherapy ranged from 11 to 51% depending on the series likely varied by physician, patient, and institutional practice [7, 23, 25]. Patients with more than one adverse risk feature were reported to have more likely to receive adjuvant radiotherapy than those with an isolated adverse or high-risk feature [7]. In our study, while 12 (25%) of the patients in the RP group received adjuvant ExRT, 9 of them also received ADT in combination with adjuvant ExRT.

Furthermore, management of local and/or BF after RP with salvage radiotherapy and ADT may permit excellent long-term outcomes comparable to definitive ExRT. In our study, while 20 (41.7%) of the patients in the RP group received salvage ExRT for local and/or BF, 12 of them received ADT in combination with salvage ExRT. In addition, the effectiveness of radiotherapy for PSA failure is greatest when given at lower levels of PSA [20]. In our study, salvage ExRT was administered at a PSA level of ≤1.5 ng/mL in 65% of the patients.

RP may reveal ascertainment errors particularly in patients who are characterized as high-risk by biopsy alone and may represent the sole, initial definitive therapy in some high-risk PCa patients with specimen-confined disease. A recent study of 1424 PCa who underwent open or robotic-assisted RP found that 61.5% (91/148) of patients with clinical GS 8 disease on prostate biopsy were downgraded on RP [32]. In our study 7 (14.6%) patients were downgraded on RP specimen from a high risk GS 8 to an intermediate risk GS 7. However, 5 of them had T3 disease with or without positive lymph node(s) on RP and the rest 2 patients had no other pathological or clinical high-risk factor except the initial GS 8 at biopsy.

Since high-risk PCa patients have high frequency of nodal spread, RP with PLND provides accurate staging to estimate prognosis and to inform the need for subsequent therapy [33]. In two systematic reviews, extended PLND had been suggested to increase the detection of positive nodes, with an associated improvement in survival [34, 35]. The observed survival benefit was attributed to the elimination of micro metastatic disease. In our study, all patients underwent RP with extended PLND and the median number of removed lymph nodes was 12 (5–38).

Radiotherapy dose escalation may provide better biochemical control in patients with high-risk disease [36–38]. Additionally, a better local control and improvement in distant metastases and disease-specific survival with higher doses of radiation are reported previously [36, 39]. However, an improvement in OS has not been demonstrated up to date. Thus, it brings to mind the question of whether the addition of long-term ADT to radiotherapy may be more important than dose escalation; most likely related to the effect of ADT on micro metastatic disease [40]. In our study, median definitive ExRT dose was 74 Gy (70–76) and 90.3% of the definitive ExRT group used ADT more than one year.

The limitations of this study are being a retrospective study, having a relatively short observation period and the use of BF as a primary endpoint since the definitions of BF are different after radiotherapy and after surgery. Furthermore, we recognize that our study is also limited by the imbalanced distributions of PSA levels and age, and the differed follow-up period between the treatment groups which could have impacted our long-term comparisons. Higher pretreatment PSA levels and older age in the definitive ExRT group than the RP group could be explained by the referral of these patients considerably to radiotherapy rather than surgery and shorter follow-up period in the RP group than the definitive ExRT group could be explained by the recently recognition of RP as an optional treatment in the context of multimodal treatment [3, 4] in high-risk PCa patients.

The strength of this study is using 3D-CRT or IMRT in all patients receiving ExRT and administration of long term ADT in most of the patients receiving definitive ExRT, and having extended PLND in all patents who underwent RP.

In high-risk PCa patients, RP as a component of multimodal therapy including radiotherapy and ADT may provide excellent oncological outcomes comparable to definitive radiotherapy. In our study, 66.7% of the patients in the RP group received either adjuvant or salvage ExRT with or without ADT and both treatment groups had similar mortality rates though the BF rate was higher in the RP group.

## Conclusion

This study showed that while the rate of BF was significantly higher and the mean BFFS was significantly lesser in high-risk PCa patients undergoing RP than definitive ExRT; 5-year DMFS, CSS and OS were similar in both treatment groups. However, further randomized trials investigating optimal management of high-risk PCa and the sub-groups are needed.

## Abbreviations

3D-CRT: Three dimensional conformal radiation therapy
ADT: Androgen-deprivation therapy
AUA: American urological association
BF: Biochemical failure
BFFS: Biochemical failure free survival
CSS: Cancer-specific survival
CT: Computed tomography
DMFS: Distant metastasis free survival
EAU: European association of urology
ExRT: External-beam radiotherapy
GS: Gleason score
IMRT: Intensity modulated radiation therapy
OS: Overall survival
PCa: Prostate cancer
PET: Positron emission tomography
PLI: Pelvic lymphatic irradiation
PLND: Pelvic lymph node dissection
PSA: Prostate-specific antigen
RP: Radical prostatectomy
